# Extended stereopsis evaluation of professional and amateur soccer players and subjects without soccer background

**DOI:** 10.3389/fpsyg.2014.01186

**Published:** 2014-10-20

**Authors:** Jan Paulus, Jie Tong, Joachim Hornegger, Michael Schmidt, Björn Eskofier, Georg Michelson

**Affiliations:** ^1^Pattern Recognition Lab, Department of Computer Science, Friedrich-Alexander University Erlangen-NürnbergErlangen, Germany; ^2^Institute of Photonic Technologies, Department of Mechanical Engineering, Friedrich-Alexander University Erlangen-NürnbergErlangen, Germany; ^3^Erlangen Graduate School in Advanced Optical Technologies, Friedrich-Alexander University Erlangen-NürnbergErlangen, Germany; ^4^Department of Ophthalmology, Friedrich-Alexander University Erlangen-NürnbergErlangen, Germany

**Keywords:** stereopsis, soccer, stereo acuity, depth perception, visual performance

## Abstract

Stereopsis is one of several visual depth cues. It has been evaluated for athletes of different types of sports in the past. However, most studies do not cover the full range of stereopsis performance. Therefore, we propose computer-supported stereopsis tests that provide an extended assessment and analysis of stereopsis performance including stereo acuity and response times. By providing stationary and moving stimuli they cover static and dynamic stereopsis, respectively. The proposed stereopsis tests were used to compare professional and amateur soccer players with subjects without soccer background. The soccer players could not perform significantly (*p* ≤ 0.05) superior than the subjects without soccer background. However, the soccer players showed significantly (*p* ≤ 0.01) superior choice reaction times for monocular stimuli. The results are in congruence with previous findings in literature.

## INTRODUCTION

Stereopsis is one of the fastest visual depth cues (Cutting and Vishton, 1995) proven to enhance the learning effect for one-handed catching (Mazyn et al., 2004, 2007) and the performance of fine motor skills (O’Connor et al., 2010a,b). Stereopsis is important in dynamic situations (Bauer et al., 2001) which require rapid visual functions. This suggests that athletes in sports such as baseball, basketball, and soccer may benefit from highly developed stereopsis. Athletes in a competitive environment are required and thus are trained to rapidly and accurately estimate the distance of the ball. Higher stereopsis performance could be assumed.

However, the significance of an athlete’s training and level of competitiveness compared to subjects who are inexperienced with a higher level of play is not fully understood as studies revealed controversial results (Abernethy et al., 1994; Boden et al., 2009). Laby et al. (2011) suggest that particular sets of visual skills like stereo acuity are sports dependent, whereas Memmert et al. (2009) conclude that highly trained athletes do not show superior basic visual skills based on basic visual measures. Therefore, these results suggest that further developments in the test methodology of stereopsis are required.

Stereopsis is typically quantified by measuring near static stereo acuity, only one component of stereopsis performance, which may not be sufficient to reliably reveal the advantages of sports vision. However, previous studies have suggested additional key components of stereopsis that may be significant in sports.

It is known that stereo acuity is degraded when the duration of the stimulus decreases (Ogle and Weil, 1958; Tyler, 1991). Therefore, recognition speed can be assessed as a qualitative factor of stereopsis performance (Saladin, 2005). Coffey et al. (1994) addressed this factor by including stereopsis response times when comparing professional golfers with amateur and senior golfers. They could reveal superior response times for professional golfers.

Another significant factor of stereopsis is the measurement of distance stereo acuity. Stereo acuity is typically measured for near distances. However, near distances might not be sufficient to fully describe stereo acuity (Bradshaw and Glennerster, 2006). Coffey and Reichow (1990) listed distance stereo acuity as an important component for sports vision. Laby et al. (1996) revealed significantly superior distance stereo acuity of major league baseball players compared to minor league players.

Independently from the distance, stereo acuity is usually measured by presenting a static stimulus. However, it was shown that there is not a significant correlation between static and dynamic stereopsis (Zinn and Solomon, 1985). Subjects who were identified to be stereo-deficient by conventional static stereo tests could make depth judgments on dynamic displays (Rouse et al., 1989). This suggests that even if static stereopsis between two groups does not reveal significant differences, dynamic stereopsis tests may reveal significant differences. The use of dynamic stereopsis may be beneficial in the prevention of accidents by integrating dynamic vision tests with standard visual tests (Sachsenweger, 1986). Dynamic stereopsis may also be more relevant in sports which utilizes a moving target and should be evaluated accordingly. Previous studies such as that of Solomon et al. (1988) have demonstrated significant differences for dynamic stereopsis between baseball players and inexperienced subjects.

The standard means of measuring stereopsis fail to reveal the specific, potential contribution of each component – recognition speed, distance stereo acuity, and dynamic stereopsis – in athletes. Therefore, we propose extended performance tests for distance stereopsis that provide static and dynamic stimuli, modeling the estimates of the recognition times as a function of presented disparities. Thus, these tests specifically assess stereo acuity and recognition speed for static and dynamic stereopsis. Given the demand and popularity of soccer in Europe, we chose to use the proposed tests to compare professional and amateur soccer players with subjects without experience in soccer. Previous studies (Ward and Williams, 2003) performed basic optometric tests including stereo acuity tests to compare the perceptual performance between elite soccer players of 9–17 years and sub-elite players of the same age. The results were not able to show a significant difference in the visual functions between the two groups. It is not clear if this is due to the inherent limitation of basic optometric tests. A focused and extended analysis of distance stereopsis in soccer is still missing that also includes speed measurements, dynamic stimuli, and a comparison between soccer players and inexperienced subjects. This study is intended to perform such measurements.

## MATERIALS AND METHODS

We developed three tests to cover stereopsis performance of athletes. (i) The monocular test is intended to assess basic choice reaction time as a baseline for the stereo tests. (ii) The static stereo test (Paulus et al., 2012a) is intended to assess static stereopsis performance as an extension to conventional static stereo acuity tests. (iii) The dynamic stereo test (Paulus et al., 2012b) is intended to assess dynamic stereopsis performance. Each test is implemented as a four alternative forced choice (4AFC) test.

### STIMULUS AND DISPLAY

All of the tests were presented on the same polarized 3D-TV (Philips 32PFL6007K/12) with a diagonal of 32 inches, a frame rate of 60 Hz, and a resolution of 1920 × 1080 pixels.

The monocular test is presented binocularly, but it is solvable monocularly. It shows one disk at one of four possible positions. The subject has to decide where the disk appears as fast as possible. This is a decision time test based on a monocular stimulus and measures a type of choice reaction time.

**FIGURE 1:**
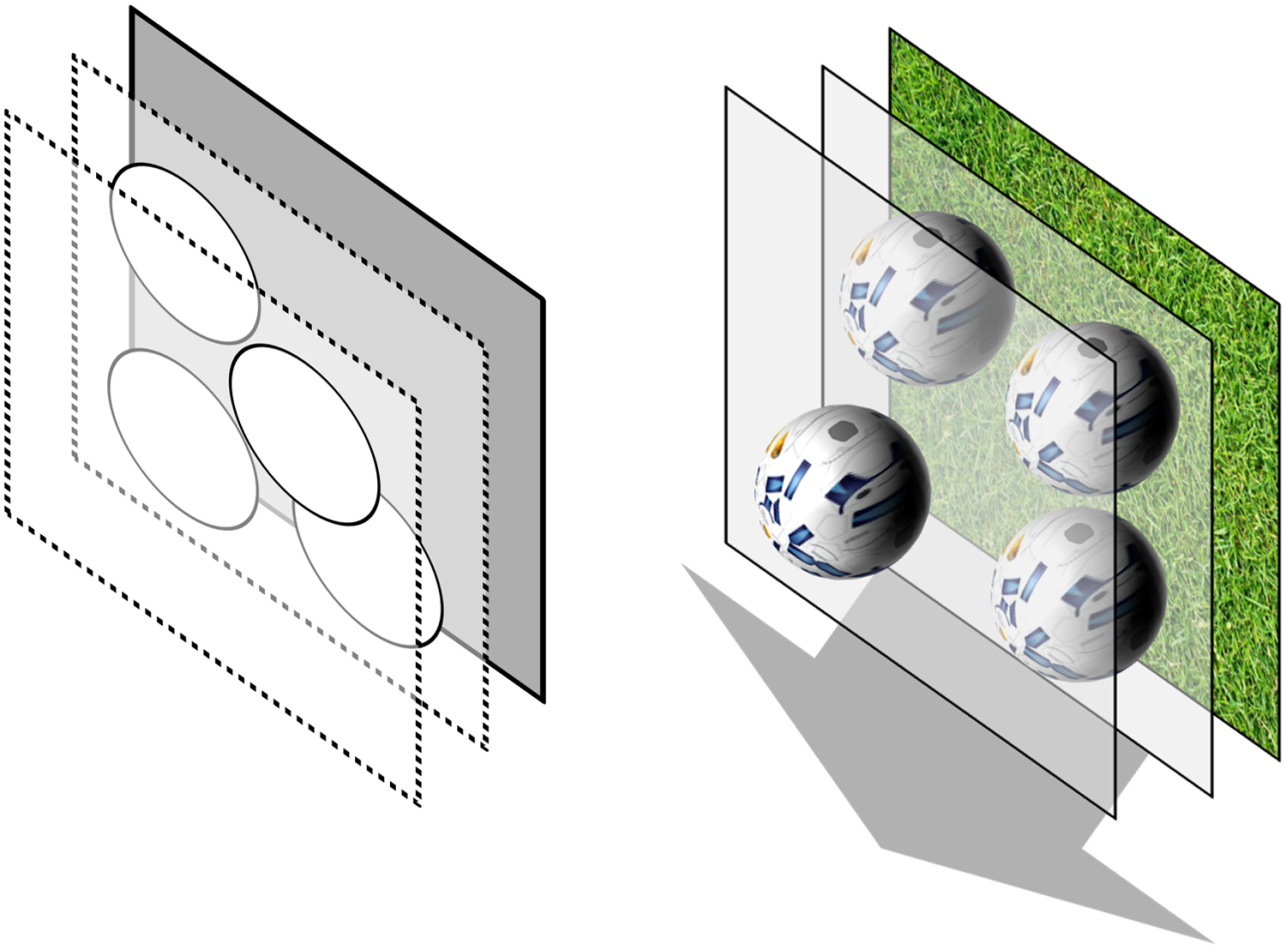
**Illustration in 3D of the static stereoscopic stimulus (left)** and the dynamic stereoscopic stimulus **(right)**. The target objects of the static test are stationary while the target objects of the dynamic test are constantly moving towards the observer.

The static stereo test provides a stereoscopic stimulus on a gray background (**Figure 1**). Four disks of the same 2D size are presented with the same disparity, further called base disparity. The base disparity of one randomly chosen disk is enlarged by a specific disparity difference such that the disk appears closer to the subject. The subject’s task is to detect this leading disk as fast as possible. The static stereo test is inspired by standard contour-based stereo acuity tests and correlates with the established Frisby test with a Pearson’s product of 0.72 (Tong et al., 2014).

The dynamic stereo test provides a moving stereoscopic stimulus on a background with grass texture (**Figure 1**). The visual targets consist of four spheres with the same soccer ball texture. Three of those virtual soccer balls are located on the screen plane; one has an enlarged disparity and appears in front of the screen plane. In this configuration, the balls move out of the screen towards the observer by continuously enlarging only their disparities. All the balls move with the same velocity. Therefore, the ball with the enlarged disparity remains appearing in front of the other balls during the whole movement. Consequently, the other balls will remain on one moving virtual depth plane during the whole movement. As the leading ball has an enlarged disparity and the disparity of all four balls is continuously increasing, the disparity difference is increasing as well. This means that when three of the balls are on the screen plane at the beginning of the movement, a certain disparity difference is set. During the movement the disparity difference is increasing as well until it reaches a maximum pre-set value. However, the observer perceives the leading ball always in the same depth difference to the other balls. Therefore, disparity difference ranges are presented. When a certain maximum disparity difference is reached, the balls are set back to their initial disparities on and in front of the screen plane, and the same procedure starts again. All four balls have the same size when observed monocularly. It continuously increases during the movement to enable a realistic impression of approaching objects. However, as the 2D size of all balls is the same at the beginning of the movement and increases in the same velocity during the movement, the 2D sizes of all balls relative to each other remain the same. This is intended to avoid an identification of the leading ball by monocular size differences. Additionally to the axial movement, each ball is equally rotating around its x-axis. The subject’s task is to detect the leading ball as fast as possible.

### INPUT

The Microsoft Kinect and its underlying pose estimation (Shotton et al., 2013) are used to enable the subjects to point into the direction of the target, which they want to select. This provides a simple and intuitive gesture control. It is intended to address the fact that the visual perceptual system and the motor system of highly trained athletes are highly connected (McLeod, 1987).

### STEREOSCOPIC PERFORMANCE DATA

We measure two components in each test and in multiple iterations to model the stereopsis performance of the subjects.

The correct decision rate estimates stereo acuity as quantitative measurement for each presented disparity difference or disparity difference range. As four disks introduce a guessing rate of 0.25 this is the lower bound of our used psychometric function (Green and Swets, 1966). This yields a psychometric threshold (PT) of 0.625. Therefore, a disparity difference or disparity difference range is classified as perceived if at least 10 out of 16 iterations were correct decisions. The probability of 10 or more correct decisions out of 16 iterations by pure guessing is lower than 0.01.

The response time estimates the recognition speed. The timer of the CPU is automatically started on stimulus presentation and stopped as soon as the subject moves one of his hands more than 30 cm away from his shoulders to indicate a target selection via the gesture control. This yields 16 response times for each presented disparity difference or disparity difference range. We compute the median of response times for correct decisions for each presented disparity difference or disparity difference range. Response times for incorrect decisions are ignored as they cannot be assumed to model the recognition speed.

### EXPERIMENTAL SETUP AND SUBJECTS

We ran a study with the proposed tests to compare the stereopsis of subjects without soccer background to the stereopsis of soccer players. The study was approved by the local ethical review board of the Friedrich-Alexander University Erlangen-Nürnberg. All subjects signed a written consent before participation.

Subjects were tested on the previously described 3D-TV at a distance of 5 m. Therefore, merely distance stereopsis was tested. Each subject performed the monocular test first, then the static stereo test and, finally, the dynamic stereo test. We presented five conventional disparity differences for the static stereo test: 15, 30, 60, 90, and 120 arcsecs. Two disparity difference ranges were used in the dynamic stereo test: 15–30 arcsecs and 60–90 arcsecs.

We measured 20 male professional and 20 male amateur soccer players of the same soccer club, the professional team, of which, plays in the German second Bundesliga. The mean age of each group was 23.6 years with a SD of 4.0 years and 19.8 years with a SD of 1.6 years, respectively. We measured all of the players in both teams. Additionally, we measured a group of 20 subjects without soccer background (“no soccer” group) and with a mean age of 29.3 with a SD of 5.3 years. The group consisted of 16 males and 4 females. None of the subjects of this group ever had constant soccer training or games in the last 5 years. Each subject of each group was tested for normal visual acuity. If subjects required additional eyewear (e.g., glasses) they had to use them during the tests. None of the subjects received stereopsis training.

### STATISTICAL DATA ANALYSIS

The three subject groups were evaluated for significant differences by using a Kruskal–Wallis test with *p* ≤ 0.05. A Wilcoxon rank-sum test (Wild and Seber, 1999), which is equivalent to a Mann–Whitney-*U* test, was conducted as *post hoc* test to identify potential significant differences between two groups. If significant differences were obtained, the procedure was repeated with *p* ≤ 0.01. Only response times for correct decisions were considered in analyses about the response times.

The monocular test was evaluated by assigning the response time median of each subject to his or her respective group. After that, the groups of response time medians were compared to each other and tested for significant differences. This was an analysis of the choice reaction time between the groups as assessed by our monocular test.

The static stereo test was evaluated in terms of static stereo acuity and response times. Stereo acuity was defined as the lowest disparity difference that a subject was able to recognize according to our used PT. Subjects who did not recognize any of the presented disparity differences (15 up to 120 arcsecs) received a stereo acuity of 180 arcsecs as this is the next higher disparity in many commonly used stereo acuity tests. The stereo acuity of each subject was assigned to the respective group of the subject. After that, the groups of stereo acuities were compared to each other and tested for significant differences. Additionally, the percentage of each group that was able to recognize a certain disparity difference according to the tested stereo acuities was computed. These calculations represented an analysis of static stereo acuity between the groups as assessed by our static stereo test. The response times were evaluated by assigning the response time median of each subject to his or her respective group. After that, the groups of response time medians were compared to each other and tested for significant differences. This was done for each disparity difference. Response time medians of subjects that were not able to recognize a disparity difference according to the used PT were not included for the respective disparity difference. This comparison was an analysis of recognition speed in static stereopsis between the groups as assessed by our static stereo test.

The dynamic stereo test was evaluated in terms of dynamic stereo acuity and response times similarly to the static stereo test. We did not test the dynamic stereo acuities between the groups for significance as only two disparity difference ranges were presented. But the percentage of each group that was able to recognize a certain disparity difference according to the tested dynamic stereo acuities was computed. This was an analysis of dynamic stereo acuity between the groups as assessed by our dynamic stereo test. The response times were again evaluated by assigning the response time median of each subject to his or her respective group. After that, the groups of response time medians were compared to each other and tested for significant differences. This was done for each disparity difference range. Response time medians of subjects that were not able to recognize a disparity difference range according to the used PT were not included for the respective disparity difference range. This comparison was an analysis of recognition speed in dynamic stereopsis between the groups as assessed by our dynamic stereo test.

## RESULTS

### MONOCULAR TEST

**FIGURE 2:**
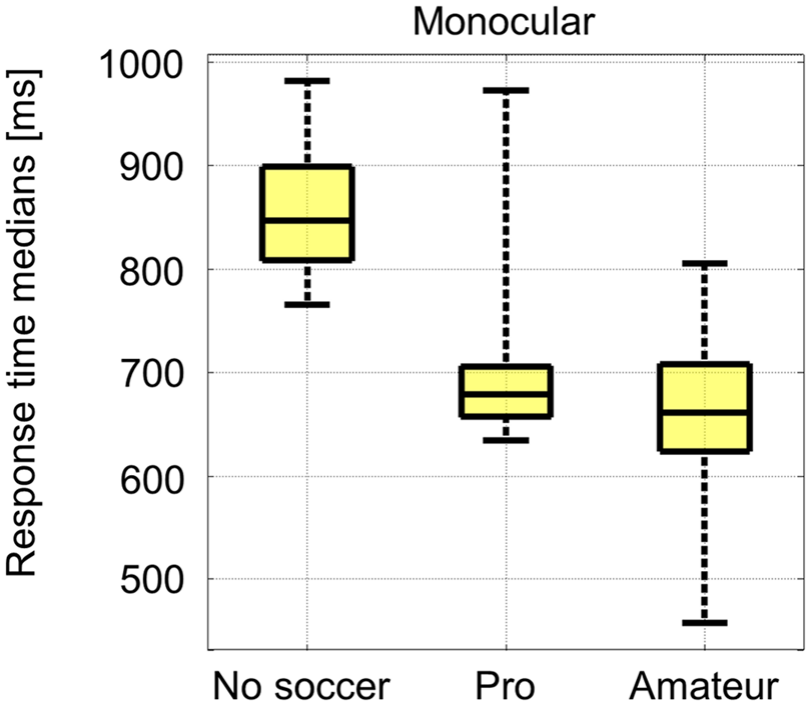
**Response time medians for the monocular task**.

The mean response time median of all subjects was 737 ms with a SD of 108 ms. The individual response time medians of the “no soccer” group were significantly (*p* ≤ 0.01) higher than the individual response time medians of each soccer group. There were no significant (*p* ≤ 0.05) differences in between the soccer groups (**Figure 2**).

### STATIC STEREO TEST

**FIGURE 3:**
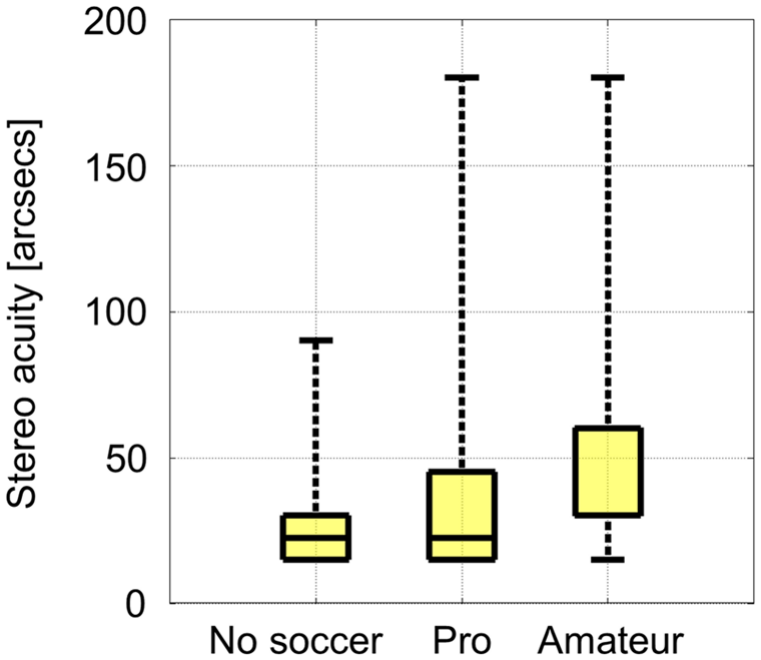
**Median of static stereo acuities per group.** The median of the amateur group corresponds to the lower bound of the box, the 25th percentile of 30 arcsecs.

The number of subjects that were able to recognize a certain disparity difference decreased with decreasing disparity differences in each group (**Table 1**). The static stereo acuities as assessed by the proposed test did not differ significantly between all groups (**Figure 3**). Also after excluding the subjects that were not able to recognize any of the presented disparity differences the static stereo acuities did not differ significantly between the groups.

**Table 1:** Percentage per group that was able to recognize a certain disparity difference or disparity difference range, respectively, according to the measured stereo acuities that are based on the used psychometric threshold (PT).

Subject group	Static					Dynamic	
	15 arcsecs	30 arcsecs	60 arcsecs	90 arcsecs	120 arcsecs	15–30 arcsecs	60–90 arcsecs
Professionals (*n* = 20)	50%	75%	85%	90%	90%	15%	75%
Amateurs (*n* = 20)	20%	60%	80%	80%	80%	65%	95%
No soccer (*n* = 20)	50%	80%	95%	100%	100%	40%	90%

**FIGURE 4:**
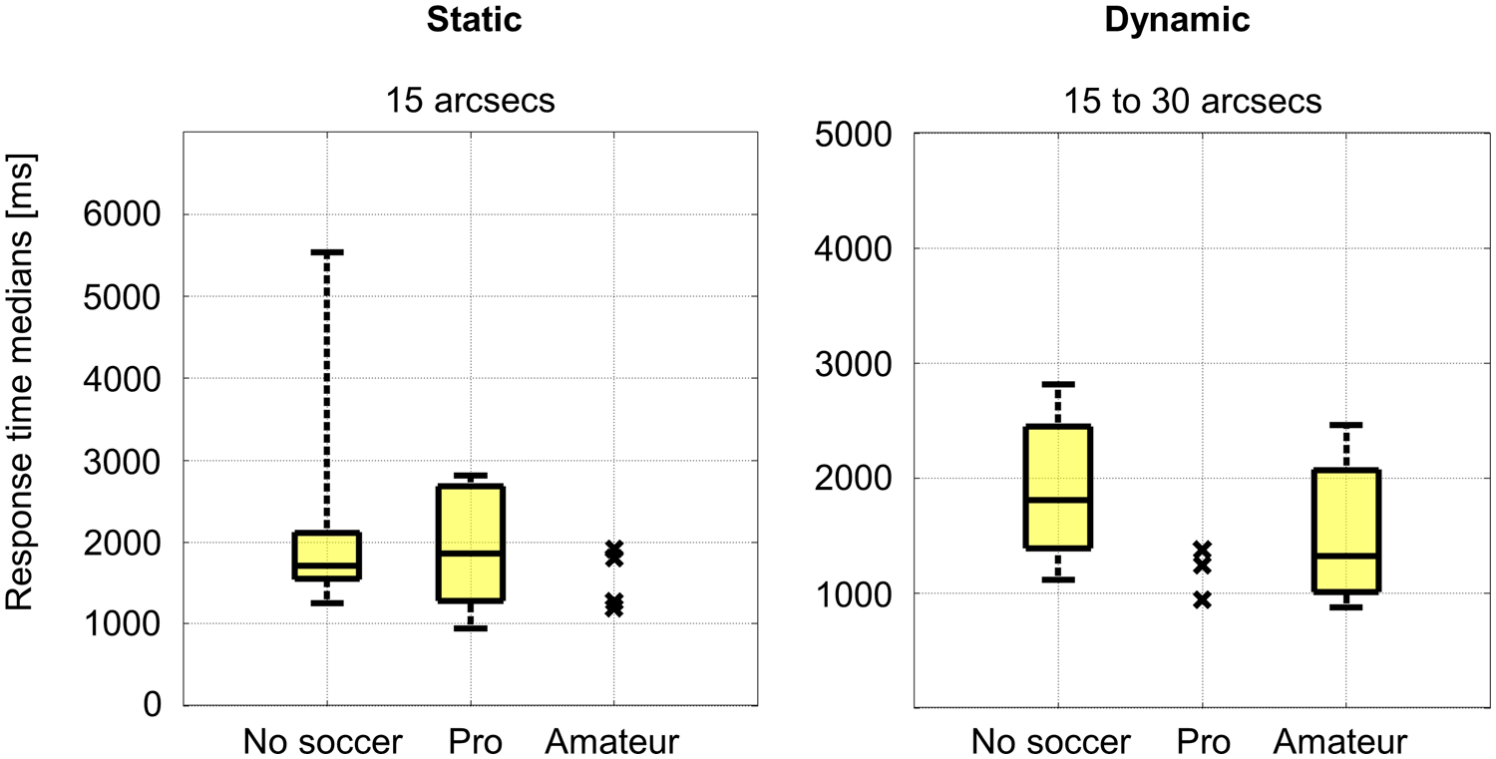
**Response time medians for the static stereo test and for the dynamic stereo test.** The results for the lowest disparity difference and the lowest disparity difference range are shown.

The mean response time median of all subjects was 1602 ms with a SD of 896 ms. The individual response time medians were not significantly (*p* ≤ 0.05) different between all groups and for each disparity difference. The results for the lowest disparity difference of 15 arcsecs are shown exemplarily in **Figure 4**.

### DYNAMIC STEREO TEST

The results were similar to the results of the static stereo test. The number of subjects that were able to recognize a certain disparity difference range decreased with decreasing disparity difference ranges in each group (**Table 1**).

The mean response time median of all subjects was 1471 ms with a SD of 659 ms. The individual response time medians were not significantly (*p* ≤ 0.05) different between all groups for each disparity difference range. The results for the lowest disparity difference range of 15–30 arcsecs are shown exemplarily in **Figure 4**.

## DISCUSSION

We expected sports such as soccer to have a high demand on stereopsis given that the sport necessitates that athletes are able to perform critically timed estimations of depth and exposes athletes to rigorous training and dynamic conditions. This initial assumption that competitive athletes could benefit from highly developed stereopsis could not be proven by the conducted study at least for soccer. The question arises if differences between soccer players and subjects without soccer background were not measurable by the used stereo tests or if, in effect, there are no differences in performance.

It is crucial to decide whether the depth information during a game is based on stereopsis or on other depth cues. Depth perception in soccer may not rely on stereopsis, which is a visual depth cue most effective for objects within 2 m, but rather on other visual depth cues such as motion perspective or relative size. For farther distances, those depth cues begin to reveal more precise depth estimations (Cutting and Vishton, 1995). Therefore, stereopsis might not be crucial for the athletes’ high performance in soccer.

However, the information provided in the proposed stereopsis tests could have had too low connections to soccer to reveal superior performance of soccer players. Dicks et al. (2010) claimed that tests have to be conducted that address the visual performance of athletes based on information that is also provided during a real game.

As the input method requires a movement of 30 cm the assumption can be made that slower recognition speed could be compensated by faster movement speeds and vice-versa. However, the response times for the monocular task can be seen as a baseline for the stereoscopic tasks. The major contribution here is the movement speed in combination with the selection time. We repeated the computations listed above by subtracting the choice reaction times of the monocular task of each subject from its stereoscopic response times. The results remained the same. Therefore, it is possible that the movement speed did not compensate for a lower recognition speed in the stereoscopic tasks. However, we cannot guarantee that the movement speed was always constant between the monocular and stereoscopic tasks. Therefore, a comparison with a button input should be evaluated in the future. Nevertheless, we assume that the input method is suitable for the evaluation of sports vision as it addresses the strong connection between the response of the visual perceptual system and the response of the motor system of highly trained sportsmen (McLeod, 1987) and as the results of the monocular test are in congruence with the literature, which suggests that choice reaction times are superior among athletes (Schwab and Memmert, 2012).

The results in the literature for static stereo acuity in different sports are controversial as presented in the introduction. However, static distance stereo acuity was identified to be superior in baseball (Laby et al., 1996) and important for sports vision (Coffey and Reichow, 1990). We evaluated distance stereopsis using an observer distance of 5 m. In our experiments we could not observe consistent differences in static distance stereo acuity as assessed by our static stereo test. However, the results as measured with the proposed static stereo test seem reasonable. The proposed static stereo test correlates with the established Frisby distance stereo acuity test with a Pearson’s product of 0.72 (Tong et al., 2014). Further, we tested the used PT by presenting a clearly visible disparity difference of 342 arcsecs to 10 subjects, who had one eye covered. None of the subjects were able to recognize the disparity difference according to the used PT. Therefore, it is reasonable that the proposed static stereo test produces valid estimations for stereo acuity and cannot be solved monocularly for the presented disparities that were clearly lower than 342 arcsecs. The presented disparity differences are also commonly used parameters in commercially available stereo tests like the TNO test (Walraven, 1975). Therefore, it seems unlikely that the static stereo task was too complex for the subjects to reveal any differences.

A comparison with the results in the literature for stereoscopic response times in sports is challenging as examples are rarely available. Coffey et al. (1994) reported superior results for professional golf players, while Solomon et al. (1988) reported superior results for professional baseball players, but only for dynamic stereopsis. We could not observe any superior results for one of the soccer groups compared to the subjects without soccer background regarding response times. One assumption could be that the proposed tests are not sensitive enough to allow the measurement of separable response time medians between the groups. But on the other hand, the test is sensitive enough to allow the observation of significantly increasing response time medians for decreasing disparity differences as the individual increase of response time medians was significant (*p* ≤ 0.01). Also the gesture control as input method is sensitive enough to reveal differences between groups for choice reactions times. If there are really differences between the groups in the response time medians for stereopsis, they are likely not as clear as the individual increase of response time medians for decreasing disparity differences or the differences in choice reaction times. On the other hand, the results of the analysis of the correct decisions rates between the groups would suggest a similar behavior regarding the response times. Therefore, if the stereo acuity of the evaluated soccer players is not superior, then response times for stereoscopic tasks may not be either.

The results of the dynamic stereo test did not reveal the same results like Solomon et al. (1988) could for baseball. Although the results of the static stereo test provided reason to expect similar results for the dynamic test, the task complexity was eventually too high to show potential differences in performance. This can be seen at the number of subjects per group that were not able to recognize the dynamic stimulus for the higher disparity difference ranges. Lower axial velocities and higher disparity difference ranges should be investigated in future studies to obtain more information.

Although our results are in contrast to studies that demonstrated superior stereopsis of athletes, those studies conducted measurements on baseball or golf, not soccer. With regard to soccer, the literature could not show a consistent discrimination in near static stereo acuity between elite and sub-elite soccer players (Ward and Williams, 2003). We did not test for near static stereo acuity in our experiments, but our results for distance stereo acuity of soccer players are in congruence with the findings of Ward and Williams (2003) for near static stereo acuity. But as a matter of fact, it cannot be stated with certainty if soccer players do not have superior stereopsis or if it could not be measured. However, this study demonstrated that the professional soccer players did not significantly perform better in the proposed stereopsis performance tests. In contrast to previous studies using standard optometric tests, the proposed tests allow a focused and extended analysis of stereopsis performance including static and dynamic distance stereo acuity in combination with response time measurements.

Although this analysis could not reveal differences between groups of soccer players, we suggest a comparison of different players by a combination of the measured values for future studies. One possible interpretation is to analyze, per subject, the static stereo acuity, the response time median at the disparity difference of the static stereo acuity and the response time median of the dynamic stereo test for 60–90 arcsecs. We assume that static stereo acuity as a quantitative measure (Saladin, 2005) has the highest importance. Therefore, we interpret subjects with lower static stereo acuities to have higher stereopsis performance. Subjects with the same static stereo acuities are compared by their response times for their static stereo threshold and their response times for the dynamic task for 60–90 arcsecs. The combination could be conducted by computing the Euclidean norm of both measures. This is a combination of the static and dynamic response times. This interpretation enables a direct comparison of different subjects by combining all stereo tests.

In conclusion, professional and amateur soccer players did not show superior results in our static and dynamic stereopsis tests compared to inexperienced subjects. However, they showed superior results on the monocular test. Therefore, our experiments could not reveal superior stereopsis performance of soccer players as assessed by our stereo tests but superior choice reaction times. The results are in congruence with previous findings about the visual performance of soccer players and extend them with measurements of distance static and dynamic stereopsis including stereo acuity and response times. The combination of our proposed tests provides a powerful tool to extensively analyze the stereopsis performance of athletes.

## Conflict of Interest Statement

The authors declare that the research was conducted in the absence of any commercial or financial relationships that could be construed as a potential conflict of interest.
